# Utilisation of malaria preventive measures during pregnancy and birth outcomes in Ibadan, Nigeria

**DOI:** 10.1186/1471-2393-11-60

**Published:** 2011-08-18

**Authors:** Olukemi O Tongo, Adebola E Orimadegun, Olusegun O Akinyinka

**Affiliations:** 1 Department of Paediatrics, College of Medicine, University of Ibadan, University College Hospital, Ibadan, Nigeria; 2 Institute of Child Health, College of Medicine, University of Ibadan, University College Hospital, Ibadan, Nigeria

## Abstract

**Background:**

Malaria remains a major public health problem in sub Saharan Africa and the extent of utilisation of malaria preventive measures may impact on the burden of malaria in pregnancy. This study sought to determine the association between malaria preventive measures utilized during pregnancy and the birth outcomes of birth weight and preterm delivery.

**Methods:**

This cross sectional survey involved 800 mothers who delivered at the University College Hospital, and Adeoyo Maternity Hospital, Ibadan. Data obtained included obstetric information, gestational age, birth weight and self reported use of malaria prevention strategies in index pregnancy.

**Results:**

Most (95.6%) mothers used one or more malaria control measures. The most commonly used vector control measures were window net (84.0%), insecticide spray (71.5%) and insecticide treated bed nets (20.1%), while chemoprophylactic agents were pyrimethamine (23.5%), Intermittent Preventive Treatments with Sulphadoxine-Pyrimethamine (IPTsp) (18.5%) and intermittent chloroquine (9.5%) and 21.7% used herbal medications. The mean ± SD birthweight and gestational age of the babies were 3.02 kg ± 0.56 and 37.9 weeks ± 2.5 respectively. Preterm delivery rate was 19.4% and 9% had low birth weight.

Comparing babies whose mothers had IPTsp with those who did not, mean birth weight was 3.13 kg ± 0.52 versus 3.0 kg ± 0.56 (p = 0.016) and mean gestational age was 38.5 weeks ± 2.1 versus 37.8 weeks ± 2.5 (p = 0.002).

The non-use of IPTsp was associated with increased risk of having low birth weight babies (AOR: 2.27, 95% CI: 0.98; 5.28) and preterm birth (AOR: 1.93, 95% CI: 1.08, 3.44). The non use of herbal preparations (AOR: 0.55, 95% CI: 0.36, 0.85) was associated with reduced risk of preterm birth. The mean ± SD birth weight and gestational ages of babies born to mothers who slept under ITNs were not significantly different from those who did not (p = 0.07 and 0.09 respectively).

**Conclusions:**

There is a need for improved utilisation of IPTsp as well as discouraging the use of herbal medications in pregnancy in order to reduce pregnancy outcome measures of low birth weight and preterm deliveries in this environment.

## Background

The burden of malaria in pregnancy (MIP) remains high in endemic areas, where despite considerable immunity, pregnant women continue to have symptomatic and asymptomatic parasitaemia resulting in adverse pregnancy outcomes. The relationship between malaria and neonatal morbidity in endemic areas such as Nigeria continues to be a subject of research. Malaria in pregnancy has been associated with significant degree of intrauterine growth restriction, 36% of preterm deliveries, 30% of preventable low birth weight deliveries, 14% of low birth weight deliveries and 15% of maternal anaemia [1]. Placental parasitaemia is associated with an estimated population attributable risk of LBW of 19% [2], abortions and still-births [3]. In view of these it is imperative to take steps to prevent malaria in pregnancy.

The World Health Organisation recommends the use of intermittent presumptive treatment with sulphadoxine pyrimethamine (IPTsp), household use of insecticide-treated bednets (ITNs) and effective and prompt case management as malaria control strategies in pregnancy [4]. Other malaria control measures recommended include personal protection measures against vectors such as use of residual sprays, window screening and mosquito repellent creams. In Nigeria, traditional remedies against malaria have always been employed, though with unproven efficacy, while chemoprophylaxis with weekly pyrimethamine and chloroquine which were widely utilised in several African countries are no longer efficacious because of emergence of resistance [5-7]. Some of the vector control measures have been proven to be effective in reducing adverse pregnancy outcomes. For instance, consistently sleeping under ITN alone has been shown to reduce the prevalence of low birth weight or preterm deliveries by more than a quarter among Kenyan mothers [8], though other studies suggested that the impact on the reduction of preterm deliveries was not significant [9]. The use of two or three doses of IPTsp has been shown to significantly lower the risk of LBW deliveries, with a relative risk of 0.51 compared with those who did not [10]. Falade and Colleagues [11] showed that birthweight of babies born to mothers who had IPTsp was higher than those who had either pyrimethamine or no chemoprophylaxis. However, the study did not evaluate other malaria preventive measures such as personal protection. Nigeria adopted the use of IPTsp as national policy in 2000. This drug as well as ITN were made available by the government and expected to be provided free to pregnant mothers during antenatal care. In order to monitor the impact of this policy, there is a need to evaluate and have information on its implementation. This study seeks to evaluate the utilisation rates of these and other malaria preventive measures by pregnant Nigerian women as well as their impacts on outcomes of pregnancy in terms of low birth weight and preterm births.

## Methods

### Study design and settings

This is a cross sectional analytical study involving 800 mothers who delivered at the labour ward of the University College Hospital (UCH) and Adeoyo Maternity Hospital (AMH), Ibadan, Nigeria over a 3 month period (November 2007 - January, 2008). The UCH is a tertiary health facility while AMH provides secondary health care in the same Ibadan North local government area, South-West of Nigeria. The two hospitals are the government-owned birth centres in the locality. ITN and IPTsp were not routinely provided free of charge at the two centres during the study period. However, there was a standard national protocol for prescribing IPTsp in place.

### Sample size

It was estimated that enrolling 733 women out of the anticipated 2000 deliveries in the two hospitals during the study period at an assumed low birthweight delivery rate of 14% (from National Demographic Health Survey data [12]) would give an estimated exact error of 2.0% at 95% level of confidence and power of 80% (using WinEpiscope 2.0 statistical software).

### Data collection

The participants were recruited consecutively during week-days at delivery and interviewed (by trained assistants) with the aid of a pretested semi-structured questionnaire within 24 hours of birth. Informed consent was obtained after explaining the study. Mothers were approached on the lying-in wards and those who declined enrolment or who delivered on weekends and had been discharged before Monday were excluded from the study. Information on maternal age, education, pregnancy history, gestational ages of babies and self reported use of 2 doses of sulfadoxine-pyrimethamine, pyrimethamine, chloroquine and herbal preparations in index pregnancy was obtained. Other data obtained included use of vector control measures such as insecticide treated bednets, insecticide spray, mosquito coils and window nets. Birth weights were recorded to the nearest 50 g using SECA weighing scales (Seca gmbh & co, Hamburg, Germany) while gestational age was obtained through mothers' last menstrual period or Ballard scores when mothers dates were uncertain and there were no early ultrasound scans available. By convention, babies born before 37 completed weeks of gestation were regarded as preterm and those weighing less than 2500 g, low birth weight. Social class stratification was based on the Oyedeji classification which is derived from the level of education and present occupation [13]. Classes 1 and 2 are regarded as upper class and 3 - 5, lower class.

### Data management and analysis

Data were recorded and analyzed with SPSS version 11 (SPSS Inc., Chicago, IL, USA). Descriptive statistics were done for all variables and also used to assess associations between pregnancy outcomes (preterm delivery and low birth weight) and malarial control measures. After the univariable analysis, multivariable logistic regression was used to further examine the association between each MIP used and outcome while controlling for the others. Effects of malaria control measures on pregnancy outcomes were presented as odds ratio (OR) and 95% Confidence interval. A *p *value less than 0.05 was considered statistically significant.

### Ethical approval

Ethical approval was obtained from the University of Ibadan/University College Hospital ethical review committee.

## Results

### Demographic characteristics

There were a total of 1220 deliveries at Adeoyo maternity and UCH during the study period. Eight hundred mothers were interviewed but 4 had incomplete data and were excluded from analysis. Of the 796 analysed, 475 (59.7%) were from Adeoyo Maternity Hospital and 321 (40.3%) from UCH. The mothers were aged between 18 and 46 years with mean (SD) of 29.4 years (5.1). Most (86.1%) were aged between 21 and 35 years while 3.3% and 10.6% were aged < 21 years and > 35 years respectively. Almost all (96.3%) were married. The majority of the mothers (78.3%) had up to 12 years of formal education, while 19.2% had less than 12 years and only 2.5% had no formal education. Other demographic characteristics are shown in table 1.

**Table 1:** Demographic details of the mothers

characteristic	Mothers (%)		
	
	UCH (321)	Adeoyo (475)	Total (796)
Mother's Age range (yrs)			

< 21	1.3	4.7	3.3

21 - 35	84.5	87.2	86.1

> 35	14.2	8.1	10.6

Level of Education			

No formal education	0.9	3.6	2.5

Primary/incomplete secondary	6.2	28.0	19.2

Secondary/Technical/Teachers Grade II Cert.	19.9	44.6	34.7

Post secondary but not university	24.9	18.5	21.0

University/Postgraduate	48.0	5.3	22.6

Social Class			

1	44.5	5.5	21.3

2	28.3	21.4	24.2

3	21.5	57.4	42.9

4	5.3	15.0	11.1

5	0.3	0.6	0.5

Marital status			

Married	99.0	99.2	99.1

Unmarried	1.0	-	0.4

Divorced/separated	-	0.4	0.3

Widow	-	0.4	0.3

The mean (SD) birthweight was 3.02 kg (0.56) and mean (SD) gestational age was 37.9 weeks (2.5). About 19.4% were preterm and 80.7% were full term; the low birthweight rate was 9.0%. Table 2 shows pregnancy outcomes by mothers' age groups and parity. There were more low birth weight deliveries among mothers less than 20 years old compared to other age groups (p < 0.05). Low birth weight risk was reduced among multigravidae and primigravidae compared with secondigravidae, while risk of preterm delivery was reduced by about half among primigravidae.

**Table 2:** Low Birthweight and Preterm delivery rates by mothers' age and gravidity

Characteristics	LBW deliveries (72)					Preterm deliveries (154)				
	
	n	% of age group	p	OR	95% CI	n	% of age group	p	OR	95% CI
Age (years)										

≤ 20*	7/26	26.9	-	1	-	7/26	26.9	-	1	-

21 - 35	55/685	8.0	0.002	0.24	0.10, 0.59	130/685	18.9	0.450	0.64	0.26, 1.54

> 35	10/85	12.0	0.117	0.36	0.12, 1.08	18/85	21.7	0.729	0.73	0.27, 2.01

No of previous pregnancies										

Primigravidae	7/92	7.6	0.240	0.56	0.24, 1.30	9/92	9.8	0.026	0.41	0.19, 0.87

Secondigravidae*	34/264	12.9	-	1	-	55/264	20.8	-	1	-

Multigravida	31/440	7.0	< 0.001	0.05	0.03, 0,08	90/440	20.5	0.981	0.98	0.67, 1.42

*reference category against which other groups were compared

P values generated from Chi sq test, and OR from 2 × 2 table analyses

### Utilisation of malaria preventive measures

Majority (95.6%) of the mothers used one or more malaria preventive measures. The most commonly used measures were window nets (84.0%), insecticide sprays (71.5%) and insecticide treated bednets (20.1%). Common chemoprophylactic agents included pyrimethamine (23.5%) and IPTsp (18.5%), while 21.7% of the mothers used traditional herbal medications. Other measures employed are shown in Table 3. The reasons given by mothers for non-utilization of prescribed malarial preventive measures were as shown in Table 4. Over 77% of the mothers who did not utilize any of the malaria preventive measures gave no specific reasons. A few of the participants reported non-affordability, non-availability, lack of belief in effectiveness of the control measures as excuses for non-utilization.

**Table 3:** Malaria preventive measures used in pregnancy

Malarial preventive measure	n	% of all 796 participants
Window nets	669	84.0

Insecticide space spray	569	71.5

Mosquito coils	333	41.8

Bed nets	281	35.3

Weekly Pyrimethamine	187	23.5

Traditional herbs	173	21.7

Insecticide treated bednets	160	20.1

Intermittent SP	147	18.5

Mosquito repellent creams	113	14.2

Intermittent chloroquine	76	9.5

SP - Sulfadoxine-Pyrimethamine. Some mothers used multiple measures

**Table 4:** Reason for non utilization of some malaria preventive measures by those who did not

Reasons	Insecticide spray (%)	Window nets (%)	Bed nets (%)	ITN (%)	SP (%)
Not affordable	3.5	2.4	5.9	5.3	4.5

Not effective/don't believe in it	2.9	1.8	1.3	2.1	1.8

I prefer other measures	7.3	4.1	10.5	10.8	14.5

Not available	0.1	1.3	5.1	7.0	0.8

No reason	86.2	90.4	77.2	75.6	78.4

A higher proportion of mothers from the upper social classes (1 and 2) utilized effective malaria preventive measures i.e. bed nets (39.1 - 43.8%), ITN (25 - 30.2%), IPTsp (20.3 - 31.4%) than mothers in the lower classes (3 - 5), (17 - 33.8%, 9.1 - 25%, 6.8 - 14.4%, respectively), whereas, the use of herbal medications was more preponderant among those in the lower social classes (31.8 - 34.1% vs 6.5 - 12.0%).

### Pregnancy outcomes

The comparison of birthweight and gestational age by malaria preventive measures utilised by participants is shown in Table 5. Only the use of IPTsp was associated with a significantly higher mean (SD) birth weight of 3.13 (0.52) kg compared to 3.0 (0.56) kg among non-users (p = 0.016). Similarly, mothers who reported the use of IPTsp had a significantly longer mean (SD) gestational age of 38.5 (2.1) weeks compared to 37.9 (0.5) weeks among non-users (p = 0.002). On the other hand, the mean (SD) birthweight of 2.88 kg (0.57) and mean (SD) GA 37.4 weeks (2.4) were significantly lower in those who used, compared with 3.10 kg (0.57) and 38.1 (2.5) (p = 0.01 and 0.001 respectively) in those who did not use herbal preparations in the index pregnancy.

**Table 5:** Comparison of mean birthweight and gestational age by malaria prevention strategies employed

Measure	Birthweight (kg)		Gestational age (weeks)	
	
	Mean (SD)	p	Mean (SD)	p
Traditional herbs				

Used	2.95 (0.51)	0.075	37.4 (2.4)	0.001

Not used	3.04 (0.57)		38.1 (2.5)	

Intermittent SP				

Used	3.13 (0.52)	0.016	38.5 (2.1)	0.002

Not used	3.0 (0.56)		37.8 (2.5)	

Intermittent chloroquine				

Used	3.06 (0.56)	0.532	38.2 (2.3)	0.370

Not used	3.02 (0.54)		37.9 (2.5)	

Weekly Pyrimethamine				

Used	3.01 (0.54)	0.620	37.8 (2.6)	0.400

Not used	3.03 (0.56)		38.0 (2.4)	

Mosquito repellent cream				

Used	3.03 (0.57)	0.838	37.8 (2.5)	0.750

Not used	3.02 (0.55)		37.9 (2.5)	

Insecticide treated nets				

Used	3.10 (0.52)	0.070	38.2 (2.2)	0.094

Not used	3.01 (0.56)		37.8 (2.5)	

Bed nets				

Used	3.04 (0.52)	0.629	38.2 (2.2)	0.190

Not used	3.01 (0.57)		37.8 (2.5)	

Window nets				

Used	3.02 (0.56)	0.881	37.9 (2.6)	0.270

Not used	3.03 (0.52)		38.1 (1.9)	

Mosquito coils				

Used	2.99 (0.53)	0.123	37.8 (2.4)	0.170

Not used	3.05 (0.57)		38.0 (2.6)	

Insecticide sprays				

Used	3.22 (0.53)	0.134	38.0 (2.4)	0.210

Not used	2.97 (0.60)		37.7 (2.6)	

Comparisons done using Students *t *test.

Mothers who reported non-usage of IPTsp had significantly higher rates of LBW and preterm births, while users of weekly pyrimethamine and traditional herbs had higher rates of preterm births. However, on multivariable analysis, non-users of IPTsp were 2 times more likely to have LBW babies (AOR: 2.27, 95% CI: 0.98; 5.28) and preterm birth (AOR: 1.93, 95% CI: 1.08, 3.44), while non-use of herbal medications was associated with reduced risk of preterm birth (AOR 0.55, 95% CI 0.36, 0.85) Table 6.

**Table 6:** Malarial preventive measures and pregnancy outcomes (low birthweight and preterm delivery)

Malarial preventive measures	Low Birthweigh (LBW) delivery						Preterm delivery					
	
	Among non-users	% of LBW among none users	Crude OR	95% CI	Adjusted OR	95% CI	Among non-users	% of preterm birth among none users	Crude OR	95% CI	Adjusted OR	95% CI
Insecticide space spray	27/227	11.9	1.57	0.95, 2.60	1.55	0.90, 2.67	54/227	23.8	1.46	1.01, 2.13	1.71	1.15, 2.56

Mosquito coils	42/463	9.1	1.01	0.62, 1.65	1.27	0.73, 2.20	85/463	18.4	0.86	0.60, 1.22	1.08	0.73, 1.59

Window nets	10/127	7.9	0.84	0.42, 1.68	0.63	0.30, 1.34	21/127	16.5	0.80	0.48, 1.32	0.64	0.38, 1.10

Bed nets	49/515	9.5	1.18	0.70, 1.98	1.23	0.67, 2.25	107/515	20.8	1.31	0.89, 1.91	1.27	0.83, 1.97

Insecticide treated bednets	59/636	9.3	1.16	0.62, 2.16	0.90	0.42, 1.94	131/636	20.6	1.55	0.96, 2.50	1.46	0.82, 2.60

Mosquito repellent creams	63/683	9.2	1.17	0.57, 2.43	1.34	0.59, 3.03	127/683	18.6	0.73	0.45, 1.17	0.59	0.35, 1.02

Weekly pyrimethamine	53/609	8.7	0.84	0.49, 1.46	0.79	0.44, 1.44	108/609	17.7	0.66	0.45, 0.98	0.72	0.47, 1.10

Intermittent chloroquine	63/720	8.7	0.71	0.34, 1.50	0.59	0.26, 1.31	141/720	19.6	1.18	0.63, 2.20	1.23	0.63, 2.42

Intermittent SP	68/649	10.5	4.18	1.50, 11.66	2.27	0.98, 5.28	138/649	21.3	2.21	1.27, 3.84	1.93	1.08, 3.44

Traditional herbs	50/623	8.0	0.60	0.35, 1.01	0.60	0.33, 1.08	106/623	17.0	0.53	0.36, 0.79	0.55	0.36, 0.85

OR: Odds ratio. All covariates shown in the table as well as maternal age and parity were included in the model for prediction of low birthweight and preterm delivery.

## Discussion

Our study showed that despite the institution of IPTsp and ITN as the national policy on MIP, only 20% uptake of ITN and 18.5% for IPTsp was documented, with significant use of pyrimethamine monotherapy, and this uptake level is well below the expected target of over 50% in the Nigerian national policy on malaria prevention strategies document [14]. The study also showed that consumption of traditional herbal medications for prevention of MIP is still a common practice among Nigerian pregnant women. These low uptakes and prevailing use of herbal remedies may have contributed to the high preterm and low birthweight deliveries demonstrated in this study. In spite of the Nigerian national policy and the relative availability of methods for preventing malaria in pregnancy, the poor uptake by mothers could be a result of poor government commitment, inadequate dissemination of information by healthcare workers, lack of knowledge by mothers and inadequate monitoring of implementation or competing priorities within tight budgetary constraints. There is a need, therefore, to ensure adequate dissemination of information to bring about attitudinal change as most of those who did not use these measures had no specific reasons for not doing so.

The adverse consequences associated with MIP [1] underscore the need for concerted efforts to ensure proper implementation of the national policy as adopted by other nations. Studies to evaluate the reasons for the apparent poor implementation need to be conducted. Compared with Falade *et al *[11], and despite a higher level of educational attainment in the present study group, twice as many mothers admitted to have used herbal preparations to prevent MIP. The higher level of education in the study group is largely accounted for by the mothers from UCH which is less patronized by people of lower socioeconomic status because of cost. Insecticide space spray which costs a lot more in terms of money and effort than ITN and SP was utilized by 71.5% of participants and was associated with a protective effect on preterm delivery but was not associated with any significant protective effect on low birthweight risk. It may give mothers a false sense of security. The fact that such a large proportion of pregnant mothers actually make the effort to protect themselves against malaria suggests that if they were better informed about the efficacy of IPTsp and ITN with accessibility and affordability, the uptake level might be much improved.

The LBW and preterm deliveries in this study, as in Dolan *et al *[15], were similar in those who slept under ITN and those who did not. Estimates for protective effectiveness of self-reported use of ITNs gave values for reduction of low birthweight at 22% (95% CI, 17.7-26.4), but lower than for SP use [16]. The present study showed that the non-utilisation of IPTsp for MIP was associated with about twice increased risk of having low birth weight babies and preterm deliveries after adjusting for the effects of maternal age and parity. Previous studies have shown that the mean birth weights were significantly higher, and incidence of LBW significantly lower, in babies born to mothers who had received two or three doses of sulfadoxine-pyrimethamine treatment than those seen in babies born to women who had had just one dose [16], and that use of three doses of IPTsp is associated with a reduction in population-attributable risk of LBW in primigravidae from 34.6% to 0% [17]. This study, as in an earlier report from Ibadan, showed that the mean birthweight and gestational age of babies delivered to mothers who used IPTsp in pregnancy were higher than any other form of prevention.

The mean birth weight was significantly higher and risk of preterm delivery reduced by half among those who did not use herbal preparations. Though the contents of the traditional medication consumed are unknown and these authors are unaware of previous documentation of the impact of traditional medications, this present study therefore highlights the need to discourage the use of traditional herbal medication through improved health education and promotion before and during pregnancy.

The financial burden of managing preterm, low birthweight deliveries in developing countries can be prohibitive compared with the cost of preventing these poor outcomes. Tongo *et al *[18] in Ibadan showed that cost of managing LBW babies can range between 22% and 3000% of combined parental monthly income where the cost of prevention of LBW and prematurity especially those associated with MIP may be as low as 1% of family income. It is therefore imperative that efforts geared towards promotion of utilization of proven effective preventive strategies should be enhanced. The commitment of government and other relevant authorities in promoting safe motherhood and roll back malaria should be enhanced by ensuring implementation of the existing policy and monitoring.

The study relied solely on self reported use of malaria preventive measures as indicator of utilization and this may have some limitations in the interpretation of the actual effects of these measures on pregnancy outcomes. Though the different social classes in the population were represented in this study, care has to be taken in extrapolating data to the whole population. The study excluded mothers who delivered on weekends, but it is not expected that the subjects' characteristics would differ whether they delivered on weekdays or weekends.

## Conclusion

Most pregnant mothers in this environment utilize malaria preventive measures. The utilization rates of IPTsp and ITN are still low while that of herbal medications is high with attendant increased risk of low birth weight and preterm births. There is a need to educate mothers on the proven benefits of IPTsp and ITN as well as ensure their availability in other to have improved pregnancy outcomes.

## Competing interests

The authors declare that they have no competing interests.

## Authors' contributions

OOT designed, conducted the study, drafted and revised the manuscript, AEO participated in the design, did data analysis and interpretation as well as revision of the manuscript, OOA conceived the study and participated in the design and revision of the manuscript. All authors have read and approved the final manuscript.

## Pre-publication history

The pre-publication history for this paper can be accessed here:

http://www.biomedcentral.com/1471-2393/11/60/prepub
